# Intervention Effect of the Mobile Phone APP Based Continuous Care on Patients after Mechanical Heart Valve Replacement: A Randomised Controlled Trials

**DOI:** 10.31083/j.rcm2509314

**Published:** 2024-09-05

**Authors:** Nana Ding, Xian Luo, Jiamei Zhou, Xue Jiang, Xiaohua Wang

**Affiliations:** ^1^Vascular Surgery Department, The Affiliated Hospital of Guizhou Medical University, 550004 Guiyang, Guizhou, China; ^2^The First Ward of the Neurosurgery Department, Affiliated Hospital of Zunyi Medical University, 563099 Zunyi, Guizhou, China; ^3^Cardiac Surgery Department, Affiliated Hospital of Zunyi Medical University, 563099 Zunyi, Guizhou, China; ^4^Nursing Department, Puding County People's Hospital, 562199 Anshun, Guizhou, China; ^5^School of Information Engineering, Zunyi Medical University, 563006 Zunyi, Guizhou, China

**Keywords:** APP, continuity care, mechanical heart valve replacement

## Abstract

**Background:**

To determine the effect of continuous care based on mobile application on patients' quality of life and self-care ability after mechanical heart valve replacement.

**Methods:**

Ninety patients who underwent cardiac mechanical valve replacement in the cardiovascular surgery department of a tertiary general hospital in Guizhou Province from September 2020 to January 2022 were selected for the study. The subjects were randomly divided into a control group and an intervention group using the SPSS25.0 software, with 45 patients in the two groups. The control group received routine nursing care during hospitalization, health education the day before discharge, and regular telephone and outpatient follow-up 6 months after discharge. Based on the control group's care, the intervention group received Mobile APP continuous care for 6 months. The effectiveness of patient's quality of life, medication adherence, international normalized ratio (INR) value compliance rates and self-care ability following mechanical heart valve replacement were evaluated the day before discharge and at the 1, 3 and 6 months after discharge.

**Results:**

Scores of quality of life, warfarin medication adherence, and self-care ability rose considerably in the intervention group compared to the control group, and the differences were statistically significant.

**Conclusion:**

The mobile phone application (APP) 's continuity of care could improve patients' quality of life, medication adherence, INR value compliance rates, and self-care ability.

**Clinical Trial Registration:**

No: ChiCTR2400081250. Registered 27 February, 2024, https://www.chictr.org.cn.

## 1. Introduction

Valvular heart disease (VHD) is a group of heart disorders in which the heart valves are narrowed or (and) do not close properly due to a variety of causes [1]. VHD is one of the leading causes of cardiovascular mortality and morbidity in the world [2]. At present, heart valve replacement (HVR) is the primary treatment for VHD [3, 4]. Heart valve prostheses used in HVR can be divided into mechanical and bioprosthetic valves. Many mechanical heart valve replacement (MHVR) patients are discharged from the hospitals with very different health behaviors than when they were in the hospital [5]. However, with prolonged discharge, patients’ self-management and treatment adherence declines, leading to insufficient or excessive anticoagulants, and complications such as embolism and bleeding occur, which can endanger patients’ lives [6, 7]. Transitional care is the extension of high-quality medical services to the family to understand the compliance behavior, treatment effect, and psychological state of the patients after discharge from the hospital and provide them with medical and psychological guidance to improve their quality of life [8, 9]. Applications (APPs) are new tools that can be downloaded to mobile devices such as tablets and smartphones. Due to the combination of APP and traditional continuous care, it can make up for the deficiency of conventional continuous care.In recent years, many scholars [10, 11, 12] have combined APP with traditional continuity care, and studies have shown that APP can meet patients’ needs for continuous care and improve the quality of continuing care and the satisfaction of nurses and patients. This study developed a “banhu” APP to provide evidence-based and scientifically authentic health education content, medical information support, and an online instant messaging function for patients after MHVR. It is convenient for patients after MHVR to carry out continuous care from admission to discharge, thus improving their quality of life.

## 2. Method

### 2.1 Design

Participants were randomly allocated to the intervention or control group in a 1:1 ratio using SPSS25.0 statistical software (Version 25.0, IBM Corp., Armonk, NY, USA) random number sequence. The allocation was kept in an opaque envelope and given to the participants according to their sequence of study entries. The research assistant who conducted randomization was uninvolved in participant recruitment, intervention delivery, and outcome assessment.

### 2.2 Patients

Ninety patients were selected for MHVR in the Department of Cardiovascular Surgery of grade 3 and first-class hospital in Guizhou Province between September 2020 to January 2022. (1) MHVR was performed for the first time under general anesthesia for patients diagnosed with VHD based on clinical manifestations and cardiac colour ultrasound; (2) age 18–65; (3) patients or their family members can effectively use the smartphone APP and are willing to cooperate; (4) at least a primary school education; and (5) patients or their guardians provided informed consent. Exclusion criteria: (1) Patients with cognitive dysfunction and mental disorders; (2) patients with severe chronic diseases (kidney, respiratory, liver failure, malignant tumour, coronary heart disease, diabetes, cerebral haemorrhage, etc.); (3) severe heart failure (cardiac function grade IV or higher); and (4) unwillingness to cooperate with the study. Drop-out criteria: (1) Patients who voluntarily withdrew during the intervention; (2) patients who passed away during the intervention; and (3) patients who did not finish the whole intervention and data collection. In the intervention group (n = 45), two individuals perished, and two persons lost to follow-up. In the control group (n = 45), one individual passed away, and another lost to follow-up. There were a total of 84 participants in the research. There was no significant difference between the groups when comparing the general information of the intervention and control groups, as shown in Table 1.

**Table 1.** **Comparison of general data between the two groups**.

Items		Intervention group	Control group	χ^2^/*t*/*Z*	*p*
Number (example)		41	43		
Gender [example (%)]				3.063^a^	0.080
	Male	19 (46.34)	12 (27.91)		
	Female	22 (53.66)	31 (72.09)		
Age (years)		51.32 ± 0.77	52.00 ± 1.08	0.515^b^	0.608
Nationality [example (%)]					0.354
	Ethnic Han	38 (92.68)	42 (97.67)		
	National minority	3 (7.32)	1 (2.33)		
Place of residence [example (%)]				0.017^a^	0.897
	City	11 (26.83)	11 (25.58)		
	Country	30 (73.17)	32 (74.42)		
Standard of culture				–0.178^c^	0.859
	Primary school	26 (63.40)	30 (69.77)		
	Junior middle school	14 (34.15)	7 (16.28)		
	High school or technical secondary school	1 (2.44)	4 (9.30)		
	Junior college	0	1 (2.33)		
	Undergraduate course	0	1 (2.33)		
Marital status [example (%)]					0.235
	Married	39 (95.12)	43		
	Divorce or separation	2 (4.88)	0		
Cardiac function [Example (%)]				–1.427^c^	0.153
	I	5 (12.20)	6 (13.95)		
	II	6 (14.63)	14 (32.56)		
	III	20 (48.78)	15 (34.88)		
	IV	10 (24.39)	8 (18.60)		
Surgical approach [Case (%)]				1.381^a^	0.501
	Mechanical aortic valve replacement	7 (17.07)	4 (9.30)		
	Mechanical mitral valve replacement	18 (43.90)	23 (53.49)		
	Mechanical double-valve replacement procedure	16 (39.02)	16 (37.21)		
Etiology				0.178^a^	0.673
	Rheumatic heart disease	21(51.22)	24 (55.81)		
	Valvular heart disease	20 (48.78)	19 (44.19)		
Ejection fraction (%)		54.90 ± 1.41	52.30 ± 1.45	–1.283^b^	0.203

Note: Comparison of ethnicity, marital status, and place of residence was performed using Fisher’s exact test, where a is the χ^2^ value, b is the *t* value, and c is the *Z* value.

### 2.3 Control Group

Patients underwent routine care during hospitalization, e.g., preoperative preparation, postoperative rehabilitation care, etc.; health education on the day before discharge; and telephone and outpatient follow-up after discharge. Post-discharge follow-up: Telephone follow-up was conducted twice in the first month after discharge, once a month after that until the end of the study, and outpatient follow-up at the first month, third month, and sixth month after discharge in conjunction with the time of the patient’s re-examination.

### 2.4 Intervention Group

The intervention group implemented mobile phone APP continuity of care on top of the control group.

### 2.5 Mobile APP Design Concept and Development

In the context of “Internet + Medicine”, the research group decided to develop a mobile phone APP specifically for post-MHVR patients after reviewing relevant literature and consulting with medical informatics experts and medical and nursing experts in cardiovascular surgery. Firstly, the researchers of the research group initially formulated the functions and content modules of the mobile phone APP, etc. Then, they consulted the cardiovascular surgery medical and nursing experts as well as medical informatics experts according to the design and setting of the functions and content modules of this mobile phone APP, discussed in depth the functions and contents design of the mobile phone APP with the patients after MHVR, and found out the needs of the patients. The design and setting of the mobile phone APP functions and content modules were improved by integrating the recommendations of medical informatics experts and cardiovascular surgery experts, as well as the needs of the patients after MHVR. Finally, we consulted and contacted the software designer and explained the mobile phone APP design concept, functions, and modules. After both parties agreed on the price and set the time for development, we tried to develop a mobile phone APP called “ban hu”, which could be accepted by post-MHVR patients and have an effect.

### 2.6 The Functions and Modules of the Patient-Side and Medical Terminal of “ban hu” APP

① Health Education Module: Patients can readily access relevant health knowledge and medical information after discharge. ② Health management module: Patients record daily indicators, including motion, heart rate, blood pressure, etc. Medical personnel may observe and provide real-time feedback via the medical terminal. ③ Medication recording module: Patients record the name, dose, use, and frequency of medications taken, as well as set ringing or vibrating medication reminders. The doctor regulates the dose of warfarin based on the patient’s international normalized ratio (INR) values at each review session, and the new dose is sent back to the patient and entered into the medication record module. The medication reminder is set every evening at 21:00 to remind the patient to take their medicine. The medication reminder is a bell or vibration, and the time clock is punched when the patient has taken their prescription. ④ Laboratory Records Module: The INR values, prothrombin time, potassium and sodium values of patients retested after discharge are manually entered into the record, and other labs are photographed and uploaded so that healthcare professionals can view them and give feedback at any time through the healthcare terminal. ⑤ Nurse-patient consultation module: Patients who encounter or have problems after discharge can consult through the interactive Q&A on Tuesdays and Fridays every week or leave a message at any time, and the nurses check the messages from time to time to give answers. And if they can’t solve the problems, they will consult the doctors in time and give the correct answers. ⑥ Review reminder module: The patients can set the review date according to the doctor’s prompts, and the reminder could be ringing or vibrating. ⑦ Personal settings: Including the patient’s name, age, gender, procedure name, etc. ⑧ Nurse-patient consultation module: Designed for patient communication. ⑨ View module: You can view the health dynamics of each module of the patients.

A group receiving ongoing care was created (1 cardiac surgeon, 2 attending nurses, 1 dietitian). During hospitalization, members of the continuity of care team explained the purpose of the study to the patients and gained the cooperation and trust of the patients and their families. Members of the continuity of care team routinely engaged in ward rounds, comprehended the progression of the patient’s condition, treatment status, postoperative rehabilitation, patients’ and family members’ level of disease-related cognition, and identified and anticipated patients’ requirements. The first day after the patients set the surgery date, the nurses assisted the patients or family members in downloading the “ban hu” APP and registering for an account, and at the same time, informed the patients or family members of the purpose of using the APP. During installation and discharge, the nurses taught the patients or their families to use the APP skillfully to improve the patient’s compliance with use. After the patients were discharged from the hospital, they could learn the contents of the health education module in the APP. If they encountered or had problems, they could consult the nurse through the APP on Tuesdays and Fridays every week or leave messages on the APP in time, and the nurse checked whether there were messages from time to time and answered them accordingly. The nurses kept abreast of each patient’s health status and, for the common problems of patients, created corresponding content in the APP to solve the patients’ problems. The nurses guided patients to read individualized health education knowledge for individual issues according to specific situations. At the same time, the nurses read the patient’s background usage data. If the patients used the APP for less than one week, the nurses contacted them by phone to inform them of the significance of actively using the APP software and advised them to continue using it. The continuity care team members followed up on discharged post-MHVR patients for 6 months via the APP.

## 3. Evaluation Tool

The standard SF-36 score questionnaire was used in this study, which includes eight scales: Physical functioning (PF), social functioning (SF), role-physical (RP), role-emotional (RE), mental health (MH), vitality (VT), bodily pain (BP) and general health (GH) [13, 14]. A Chinese-translated version of the standard SF-36 questionnaire was used for practical purposes.

The study [15] revealed that the Cronbach’s alpha coefficient for the Chinese version of the Morisky medication compliance Scale 8 was 0.81, the consistency coefficient across raters was 0.92, the retest correlation coefficient was 0.95, and the surface validity was good, with a content validity of 1.00. The scale has eight questions and a single dimension. The options of questions 1 through 7 are dichotomous, i.e., “yes” and “no”; the answer “no” counts for 1 point, the answer “yes” counts for 0 points, the fifth reverse score, and the eighth question options for Likert 5 rating, i.e., “never”, “rarely”, “sometimes”, “often”, and “always”, score 1 point, 0.75 points, 0.5 points, 0.25 points, and 0 points respectively. The maximum score on the scale is eight, with higher numbers indicating greater cooperation.

The Self-Care Competency Rating Scale was adopted from Lina Guo *et al*.’s [16] modified version of the 2014 Chinese version. The questionnaire’s Cronbach’s coefficient is 0.77, while the expert evaluation method’s content validity is 0.87. The scale consists of 15 items, which may be separated into three dimensions: General self-care needs, developmental self-care needs, and inadequate health self-care needs.

The patients’ coagulation index INR was collected. The patients’ INR value was recorded when the patients were examined regularly, and an INR of 1.5 to 2.5 was regarded as the INR reaching the standard [17]. The INR meeting rate of patients in this group was obtained by calculating the ratio of the number of patients meeting the INR standard to the total number of patients in this group.

## 4. Data Collection and Entry

Before the start of the study, the continuity of care team members underwent standardized training, where the researcher’s assistant talked to them about the purpose of the study, the methodology, content, and significance of the intervention, and each member was well aware of the use of the various assessment tools and questioning techniques. The continuity of care team members were responsible for implementing the intervention. To avoid contamination and interference, patients in the intervention and control groups were assigned to different rooms in consultation with the bedside doctor and the head nurse, and the patients agreed to maintain confidentiality for their participation in this study to prevent patients from influencing each other. The research assistants responsible for data collection were blinded to group assignments. The day before discharge, the general information, standard SF-36 score questionnaire, Morisky medication adherence scale, and self-care capacity evaluation scale were gathered. Patients’ indicators were recollected 1, 3, and 6 months after discharge through telephone, outpatient follow-up, or the “ban hu” APP. Two individuals input and double-checked every data.

## 5. Statistical Method

For statistical analysis, SPSS 25.0 was employed as a statistical programme. Normally distributed measurement data were written as $(\bar{x}$ ± s) using the independent sample *t*-test. Non-normally distributed measurement data were expressed as M (QR) using the Wilcoxon rank sum test. A 2-tailed significance level of 0.05 was used.

## 6. Results

On the day before discharge, the intervention and control groups had no significant difference in the SF-36 scale, Morisky medication adherence scale, and self-care competence assessment scores. At 1 month after discharge, the intervention group had higher scores than the control group on the PF, GH, VT, SF, RE, and MH dimensions; at 3 and 6 months after discharge, the intervention group scored higher than the control group on all dimensions of the SF-36 scale, and the difference was statistically significant (Table 2). At 1, 3, and 6 months after discharge, the intervention group had higher scores than the control group on adherence to warfarin medication and evaluation of self-care skills; the difference was statistically significant (Tables 3,4).

**Table 2.** **Comparison of quality of life between the two patient groups ($\bar{x}$ ± s, score)**.

Items	Grouping	The day before discharge	Post-discharge		
			1 month	3 months	6 months
PF	Control group	13.95 ± 1.33	36.28 ± 2.06^▲^	53.49 ± 2.45^▲⁢#^	62.56 ± 1.89^▲⁢#⁢✩^
	Intervention group	14.27 ± 1.45	43.05 ± 1.61^▲^	62.56 ± 1.37^▲⁢#^	82.32 ± 1.13^▲⁢#⁢✩^
	*t*	–0.161	–2.575	–3.236	–8.984
	*p*	0.873	0.012	0.002	<0.001
RP	Control group	40.12 ± 4.57	40.12 ± 4.86	41.86 ± 4.22	45.93 ± 4.4
	Intervention group	43.29 ± 4.28	51.22 ± 4.78	54.88 ± 4.96	73.17 ± 2.81^▲⁢#⁢✩^
	*t*	–0.506	–1.627	–2.007	–5.218
	*p*	0.614	0.107	0.048	<0.001
BP	Control group	39.65 ± 1.96	42.14 ± 2.13	45.98 ± 1.73	57.63 ± 1.93^▲^
	Intervention group	36.73 ± 1.74	43.05 ± 1.61	52.59 ± 2.42^▲⁢#^	68.29 ± 1.74^▲⁢#⁢✩^
	*t*	1.113	–0.338	–2.24	–4.089
	*p*	0.269	0.736	0.028	<0.001
GH	Control group	54.65 ± 1.68	59.30 ± 1.64	62.56 ± 1.92^▲^	69.07 ± 1.90^▲⁢#⁢✩^
	Intervention group	55.00 ± 1.25	65.24 ± 1.54^▲^	68.78 ± 1.63^▲^	76.95 ± 1.80^▲⁢#⁢✩^
	*t*	–0.167	–2.631	–2.46	–3.009
	*p*	0.868	0.01	0.016	0.003
VT	Control group	43.14 ± 1.97	54.30 ± 1.97^▲^	58.84 ± 1.77^▲⁢#^	57.79 ± 1.66^▲⁢#^
	Intervention group	42.80 ± 2.28	59.76 ± 1.48^▲^	64.51 ± 1.98^▲^	71.59 ± 1.69^▲⁢#⁢✩^
	*t*	0.111	–2.195	–2.142	–5.83
	*p*	0.912	0.031	0.035	<0.001
SF	Control group	50.00 ± 2.88	58.72 ± 2.58	65.99 ± 2.13^▲⁢#^	72.67 ± 2.57^▲⁢#⁢✩^
	Intervention group	50.00 ± 1.95	65.55 ± 1.99^▲^	73.17 ± 1.93^▲⁢#^	82.32 ± 2.39^▲⁢#⁢✩^
	*t*	0	–2.094	–2.488	–2.74
	*p*	1	0.039	0.015	0.008
RE	Control group	30.23 ± 5.69	43.41 ± 5.16	62.79 ± 3.55^▲⁢#^	88.37 ± 2.91^▲⁢#⁢✩^
	Intervention group	34.15 ± 5.82	58.54 ± 5.29^▲^	78.86 ± 3.63^▲⁢#^	99.19 ± 0.81^▲⁢#⁢✩^
	*t*	–0.481	–2.046	–3.196	–3.579
	*p*	0.632	0.044	0.002	0.001
MH	Control group	51.53 ± 2.09	60.00 ± 1.59^▲^	64.74 ± 1.21^▲⁢#^	67.53 ± 1.60^▲⁢#⁢✩^
	Intervention group	48.88 ± 1.15	66.63 ± 1.31^▲^	69.56 ± 1.18^▲^	75.41 ± 1.11^▲⁢#⁢✩^
	*t*	1.113	–3.204	–2.836	–4.035
	*p*	0.27	0.002	0.006	<0.001

Note: ▲ represents the day before discharge comparison, *p* < 0.05; # stands for comparison at 1 month after discharge, *p* < 0.05; ✩ stands for comparison at 3 months after discharge, *p* < 0.05. PF, Physical functioning; SF, social functioning; RP, role-physical; RE, role-emotional; MH, mental health; VT, vitality; BP, bodily pain; GH, general health.

**Table 3.** **Comparison of warfarin medication adherence between the two patient groups ($\bar{x}$ ± s, score)**.

Grouping	The day before discharge	Post-discharge		
		1 month	3 months	6 months
Control group	5.13 ± 0.22	4.89 ± 0.19	4.49 ± 0.17	4.14 ± 0.23
Intervention group	5.12 ± 0.23	5.69 ± 0.19	6.43 ± 0.18^▲⁢#^	9.29 ± 0.23^▲⁢#⁢✩^
*t*	0.012	–2.999	–7.873	–15.894
*p*	0.991	0.004	<0.001	<0.001

Note: ▲ represents the day before discharge comparison, *p* < 0.05; # stands for comparison at 1 month after discharge, *p* < 0.05; ✩ stands for comparison at 3 months after discharge, *p* < 0.05.

**Table 4.** **Comparison of self-care ability evaluation between the two patient groups ($\bar{x}$ ± s, score)**.

Grouping	The day before discharge	Post-discharge		
		1 month	3 months	6 months
Control group	44.51 ± 0.82	48.19 ± 0.50^▲^	46.67 ± 0.89	45.88 ± 0.76^#^
Intervention group	45.98 ± 0.42	50.12 ± 0.32^▲^	52.85 ± 0.52^▲⁢#^	57.07 ± 0.41^▲⁢#⁢✩^
*t*	–1.553	–3.249	–5.905	–12.742
*p*	0.122	0.002	<0.001	<0.001

Note: ▲ represents the day before discharge comparison, *p* < 0.05; # stands for comparison at 1 month after discharge, *p* < 0.05; ✩ stands for comparison at 3 months after discharge, *p* < 0.05.

There was no significant difference between the INR compliance rates on the day preceding discharge. After 1, 3, and 6 months following discharge, the intervention group had more excellent INR compliance rates than the control group (Table 5).

**Table 5.** **Comparison of the international normalized ratio compliance rates between the two groups (example %)**.

Grouping	The day before discharge	Post-discharge		
		1 month	3 months	6 months
Control group (example)	34 (79.07)	22 (51.16)	19 (44.19)	18 (41.86)
Intervention group (example)	33 (80.49)	30 (73.17)	33 (80.49)	35 (85.37)
χ ^2^	0.26	4.311	11.728	24.838
*p*	0.872	0.038	0.001	<0.001

## 7. Discussion

While the quality of life of patients following MHVR was much better than before surgery, it remained inferior to that of the general population [18]. As a form of telemedicine application that can be used regardless of geographical and time constraints, APP enables patients to access services provided by healthcare professionals promptly [19]. Therefore, as long as patients have a smartphone with internet access, they can have comprehensive access to the care methods, disease-related knowledge, rehabilitation exercises, etc., that healthcare professionals have summarised after extensive research, which complements the traditional continuity of care model [20]. This study revealed no significant difference between the two groups in terms of quality of life the day before discharge. But 6 months after discharge, the quality of life of the intervention group was significantly higher than that of the control group. Patients encountered problems at different stages of rehabilitation, so they could ask the nurses questions through the APP to get specialized help on time. Nurses help patients set up medication reminders and review dates through the APP. They encourage patients to take warfarin and review INR values on time to reduce anticoagulation-related complications. Application of APP continuity of care ensured continuity of care and treatment after discharge, to develop patients’ self-care ability, and to promote patients’ active participation in disease management, thus improving their quality of life.

Warfarin, an oral vitamin K antagonist, is the drug of choice for anticoagulation in patients after MHVR [21]. However, warfarin has a very narrow therapeutic window and a long half-life [22], and medications, lifestyle habits (e.g., alcohol consumption), mood, and diet easily influence the effectiveness of anticoagulation. In case of poor anticoagulation, serious complications (thromboembolism and bleeding) can occur and even be life-threatening [23]. Thus, improving adherence to warfarin medication and INR value compliance rates are beneficial to the long-term prognosis of patients after MHVR. This study showed that at 6 months after discharge, the medication compliance of warfarin and the INR value compliance rates in the intervention group were also more significant than those in the control group. It indicates that mobile APP continuity of care can effectively improve patients’ anticoagulation compliance and INR value compliance rates after MHVR, which was similar to the results of the study by Zhang YC *et al*. [24] and Pan QL *et al*. [23]. This was due to the one-on-one communication guidance regarding the patient’s situation. The nurses provided detailed and individualized information through the APP to improve patients awareness of anticoagulant treatment after MHVR. The APP could set up a daily timer to remind patients to take warfarin in time and a review date to remind patients to review the INR on time to adjust the dosage of warfarin. These practices can ensure that intervention group patients have a high level of adherence to warfarin medication and INR value compliance rates.

Self-care is the process by which an individual’s conscious behavioral change to maintain their health [25]. This study’s results indicated that the control group’s capacity to care for themselves was inferior to that of the intervention group. A study showed that only 60% of the patients could remember the health education given by doctors and nurses before discharge, and 82.7% of the patients wished to continue receiving professional guidance from doctors and nurses on observing their conditions and knowledge of self-care after discharge. Post-MHVR patients have multiple and complex post-discharge-related treatments due to their unique characteristics. A survey by Li SY [26] showed that 88.68% of patients, after MHVR, needed knowledge about anticoagulation therapy, and 66.04% of patients indicated that their access to relevant knowledge was through a mobile phone APP. The intervention group was discharged from the hospital with access to appropriate professional expertise and guidance through the APP, which improved their nursing competence.

The price of the APP developed in this study is reasonable, and the shareability, deliverability, and easy storage of the app platform play a vital role in reasonably utilizing medical resources, increasing the fairness and accessibility of healthcare services. Therefore, applying a mobile APP for patient tracking, follow-up, education, and adjustment of treatment plans can also save patients a lot of time and financial expenses.

## 8. Limitations

There are several limitations in this study. First, the APP developed in this study is in the preliminary application stage, and the functional modules must improve. Second, the sample size was relatively small due to the constraints of personnel, time, and other factors; only 90 patients were finally included. Third, the present study was a single-center study, and more studies must be conducted in multiple centers to assess the value of continuity of care. Fourth, during the follow-up period, the coagulation function review is performed at the local hospital, and the INR measurement method may vary from hospital to hospital. The follow-up period of this study was brief, and a long-term follow-up period is needed. The follow-up period should be extended to observe long-term anticoagulation effects better.

## 9. Conclusion

This study showed that the mobile APP transitional care can enhance the quality of life, the medication compliance of warfarin, the INR value compliance rates, and the self-care ability in patients after MHVR.

## Data Availability

All data points generated or analyzed during this study are included in this article and there are no further underlying data necessary to reproduce the results.

## Abbreviations

APP, application; VHD, valvular heart disease; HVR, heart valve replacement; MHVR, mechanical heart value replacement; INR, international normalized ratio; PF, Physical functioning; SF, social functioning; RP, role-physical; RE, role-emotional; MH, mental health; VT, vitality; BP, bodily pain; GH, general health.
